# The impact of parental migration on left-behind children’s vision health in rural China

**DOI:** 10.1186/s12889-022-14962-4

**Published:** 2023-01-02

**Authors:** Kang Du, Tianli Yang, Jin Zhao, Hongyu Guan

**Affiliations:** 1 School of Economics, Xi’an University of Finance and Economics, Xi’an, China; 2 School of Labor and Human Resources, Renmin University of China, Beijing, China; 3 School of Marxism, Xi’an University of Finance and Economics, Xi’an, China; 4 Center for Experimental Economics in Education, Shaanxi Normal University, Xi’an, China

**Keywords:** Parental migration, Left-behind children, Vision health, Myopia, Eyeglasses

## Abstract

**Background:**

Parental migration is an important factor affecting left-behind children’s health. However, few studies have addressed the effect of parental migration on children’s vision health in China. To fill the gap, this study aimed to assess the impact of parental migration on left-behind children’s vision health and to explore the possible mechanisms of the effect.

**Methods:**

Data were obtained from the baseline survey of the China Education Panel Survey (CEPS), which included over 10,000 junior high school students. This study used myopia, the most common vision problem among junior high school students, and tried to analyze whether myopia was corrected with eyeglasses as indicator variables of vision health. The impact of parental migration on vision health was assessed using an instrumental variables approach.

**Results:**

The results show that parental migration reduced the likelihood of myopia in left-behind children and decreased the possibility of myopic left-behind children being corrected. This result passed a series of robustness tests. The mechanism analysis indicated that compared to non-left-behind children, left-behind children spent more time on outdoor activities and less time on after-school classes, reducing their risk of being myopic. Further, because left-behind children live apart from their parents, their myopia problem is more difficult for parents to notice, and left-behind children are less likely to inform their parents of their myopia than non-left-behind children actively. This helps to explain why left-behind children have a lower correction rate with eyeglasses.

**Conclusions:**

Our findings suggest that parental migration, while not increasing the prevalence of myopia in left-behind children, has led to inequity in myopic left-behind children’s correction. Given the severe consequences of uncorrected myopia, action is required to enhance the correction rate of myopic left-behind children.

## Introduction

A considerable number of laborers in low- and middle-income countries migrate abroad or within the country in search of better job opportunities (e.g., from rural to urban areas) to enhance their families’ economic condition [1]. As a result of migration, children are often left behind in the care of other family members or caregivers [2, 3]. It is estimated that 27% of children in the Philippines, 36% in Ecuador, and more than 40% in rural South Africa are left behind [4]. China, the largest middle-income country, has a substantial and potentially vulnerable subpopulation of left-behind children (LBC) in rural areas. Over the last 40 years, many Chinese parents have moved from rural to urban areas in search of higher-paying jobs to increase family income [5]. Concurrently, China’s strict domestic migration policies (*hukou*) prevent these migrants from migrating with their children [6]. This leads to more than a third of all children residing in rural China (61 million) as “left behind” with one or both parents migrated [7].

There is a great concern about the impact of parental migration on the health of LBC, particularly the disparities in health outcomes and health-seeking behaviors between LBC and non-LBC. Nevertheless, the evidence is mixed. On the one hand, there is much evidence of inequity in health outcomes between LBC and non-LBC. Because of separation from their parents, mental health or health conditions that are not urgent or have few visible symptoms of LBC are more likely to be overlooked [8, 9]. For example, many studies suggest LBC have a greater risk of mental health difficulties, decreased non-cognitive ability, and suicidal ideation [10–13]. On the other hand, the material benefits of remittances and greater income security may lead to the improved health status of LBC and facilitate payment for health care services. In Pakistan, migration had positive effects on the growth of LBC [14]. Several studies in China also find that LBC has lower rates of soil-transmitted helminth infection and refractive error than non-LBC [3, 15, 16]. Thus, the effects of parental migration might vary according to the areas of health.

Vision health is an important component of adolescent health. Still, the high prevalence of myopia poses a serious threat to adolescent vision health in East and Southeast Asia regions, especially the Chinese mainland [17]. The prevalence of adolescents with myopia in rural China ranges from 24% in primary schools to more than 50% in junior high schools [18, 19]. Studies have shown that uncorrected myopia negatively affects school-aged children’s academic performance, physical and mental health, and quality of life [18, 20, 21]. Fortunately, most myopia can be easily and safely corrected by accurately prescribed eyeglasses [22]. However, the correction rate in rural areas in China is very low [23]. According to Yi et al. (2015), more than 85% of primary students in rural China with myopia do not wear eyeglasses [18]. The high prevalence of myopia and low correction rate among children in rural China lends itself to research on the effects of parental migration on children’s myopia and health-seeking behaviors (eyeglasses).

Several studies have analyzed the relationship between parental migration and students’ visual health. A study by Zhou et al. (2015) found that children living with both parents had a higher prevalence of refractive error (17%) than LBC (13%), which was significant at the 1% level [3]. Based on multiple regression analysis, Yi et al. (2015) found that children with both migrant parents were less likely to have poor vision than children whose parents lived and worked at home [18]. However, little is known about the effects of parental migration on the vision health of LBC, particularly in myopia and the correction of eyeglasses. Only one study assessed the impacts of parental migration on myopia using a propensity score matching (PSM) approach [16]. According to this study, left-behind children have lower rates of myopia than non-migrant children. However, this paper might not be representative due to the sample selection. Moreover, to our knowledge, no studies have examined whether or how parental migration affects health-seeking behaviors for caregivers of LBC and non-LBC. In China, anti-myopia campaigns have been issued since 2018 to reduce the prevalence of myopia among children, including policies on increasing outdoor activities, reducing academic burdens, and providing timely corrective treatment [24]. If parental migration leads to inequalities in health-seeking behavior for myopic children, then some action to eliminate these inequalities is needed. Therefore, studying the effect of parental migration on children’s vision health will not only help to solve the practical problem of myopia prevention and control among adolescents but also help to enrich the research on the health impacts of parental migration.

Theoretically, parental migration may have positive and negative effects on vision health (myopia and eyeglasses ownership) in LBC. This difference mainly comes from the lack of attention and supervision caused by the absence of parents. In the case of myopia, outdoor activity and near work (such as reading and writing) are major factors affecting myopia. The more time students spend on outdoor activities and less time on near work, the less likely they are to be myopic [25–27]. Therefore, if LBC spend less time on their study and more time on outdoor activities because of the absence of parents, they are less likely to be myopic [16]. Accordingly, LBC might spend more time on electronic devices (near work), thus, are more likely to be myopic. Thus, empirical research is required to determine the effects and mechanisms of parental migration on myopia among LBC. As for the healthcare service of eyeglasses correction, parents may be able to afford eyeglasses for their myopic children because of the migration. On the contrary, myopia in LBC children may be more likely to be neglected by their caregivers’ because it is not acute or has few obvious symptoms [9, 28]. It is difficult for parents of LBC to detect their children’s myopia directly, and children may be reluctant to tell their parents about it because of parent-child separation. Hence, the effect of parental migration on students’ myopia and healthcare uptake (eyeglasses) is also unclear, and further empirical studies are needed.

The main hurdle in conducting ideal research on this topic is the problem of endogeneity. Omitted variables correlated with migration and children’s vision health may cause problems. To address this problem and the research gap, we assessed the effect of parental migration on LBC’s vision health using class-level migration rates as the main instrumental variable based on data from the China Education Panel Survey (CEPS).

Specifically, we pursue three objectives. First, we describe the prevalence of myopia and eyeglasses ownership and examine the differences among subgroups based on parental migration status. Second, we estimate the impact of parental migration on LBC’s myopia and eyeglasses ownership. At last, we explore possible ways that parental migration influences children’s myopia and eyeglasses ownership. The remaining paper is structured as follows: We introduce the data and describe the variables. We then provide the econometric models and identification strategies, followed by the estimation results and discussion. Finally, we summarize the findings and suggest policy implications.

## Method

### Data

The data was drawn from China Education Panel Survey (CEPS), a large-scale survey targeting Chinese students attending 7th and 9th grades in 2013.^1^ The CEPS survey, permitted by the ethics committee of the Renmin University of China, obtained the written consent of all students and their legal guardians or next of kin. In the baseline survey year (the 2013–2014 school year), based on a multi-stage probability-proportional-to-size strategy, 28 counties/districts in China were first selected using the average education level and proportion of migrants as stratifying variables. Four schools from each chosen county/district were randomly selected using enrollment size and school type as stratifying variables. Finally, 438 classes were randomly selected in these schools, and approximately 20,000 students were surveyed. The CEPS administered separate questionnaires to sampled students, parents, teachers and school administrators. The rich information that CEPS contains facilitates our examination of the effect of parental migration on vision health.

Although follow-up surveys were conducted afterwards, our analysis focuses on data from the baseline survey because it has a larger sample size, as only 7th graders were followed in the follow-up surveys. According to the purpose of our study, we have restricted our sample to rural children. Due to missing information on key variables (i.e., migration status and vision health), the size of the final analytical sample is 10,361, with 2751 LBC and 7610 non-LBC.

### Variables

In this study, myopia and eyeglass ownership were employed as indicators for the vision health of interest. Specifically, we constructed two dummy variables to measure whether a student was myopic or owned eyeglasses (=1 if yes; = 0 if no). Firstly, students were asked whether they had myopia. We define a student is myopic if their answer is “Yes” and not myopic if the answer is “No”. Secondly, all sampled students who reported being myopic either further reported a specific refractive error (in diopters) of their eyeglasses prescription or responded that they were “unsure” about their refractive error. Based on these responses, a student was deemed to be “owned glasses (Yes=1)” if he/she reported a specific refractive error, and “not owned eyeglasses (No=0)” if he/she was “unsure” about their refractive error.

The key independent variable is parental migration, which is a value of 1 if any parent is migrated (including both parents) and 0 if both parents are at home. Meanwhile, this is also the definition of LBC in this paper. That is, if any parent is migrated, they are considered as LBC.

In addition to parental migration, LBC’s vision health (myopia and eyeglasses ownership) could be affected by many other factors. We control a set of variables in our empirical models to avoid bias from omitted variables. Control variables included demographic and family characteristics of the students in the present study, including age, gender, grade, number of siblings, school boarding, academic score, father and mother educational attainment, family economic condition, and living area. Students reported all these covariates. Table 1 presents descriptive statistics for these key variables.

**Table 1:** Summary statistics of background characteristics

Variables, mean (SD)	Full Sample (1)	Left-behind (2)	Non-Left-behind (3)	Difference: (3)–(2) ***P***-Value (4)
**Panel A: Outcome variables**				
Myopia (1 = yes; 0 = no)	0.546 (0.498)	0.512 (0.500)	0.558 (0.497)	0.046***
Owned eyeglasses (1 = yes; 0 = no) ^a^	0.407 (0.491)	0.366 (0.482)	0.420 (0.494)	0.054***
**Panel B: Child Characteristics**				
Age (years)	14.228 (1.317)	14.238 (1.377)	14.225 (1.295)	−0.013
Boys (1 = yes; 0 = no)	0.521 (0.500)	0.540 (0.498)	0.514 (0.500)	− 0.026**
9th grade (1 = yes; 0 = no)	0.484 (0.500)	0.467 (0.499)	0.490 (0.500)	0.024**
Number of siblings	0.965 (0.803)	1.075 (0.834)	0.925 (0.788)	−0.150***
School boarding (1 = yes; 0 = no)	0.474 (0.499)	0.588 (0.492)	0.432 (0.495)	−0.156***
Academic score (standardized)	−0.003 (0.995)	−0.039 (1.002)	0.010 (0.992)	0.049**
**Panel C: Family Characteristics**				
Father’s educational attainment (years)	8.995 (2.271)	8.743 (2.235)	9.085 (2.277)	0.342***
Mother’s educational attainment (years)	8.152 (2.801)	7.737 (2.960)	8.302 (2.726)	0.565***
Family economic condition:				
Poor	0.272 (0.445)	0.342 (0.474)	0.247 (0.431)	−0.095***
Average	0.686 (0.464)	0.629 (0.483)	0.706 (0.456)	0.077***
Rich	0.042 (0.201)	0.029 (0.169)	0.047 (0.211)	0.018***
Living area:				
Eastern China	0.499 (0.500)	0.299 (0.458)	0.571 (0.495)	0.272***
Central China	0.285 (0.452)	0.410 (0.492)	0.241 (0.428)	−0.169***
Western China	0.216 (0.411)	0.291 (0.454)	0.188 (0.391)	−0.103***
N	10,361	2751	7610	

^a^5660 myopic students answered whether they owned eyeglasses, including 1410 LBC and 4250 non-LBC. Column (4) shows the t-test of the difference between LBC and non-LBC

** p < 0.05, *** p < 0.01

### Statistics analysis

This study first summarized descriptive statistics of student and family background characteristics. Moreover, group comparisons of these characteristics between LBC and non-LBC were performed by sample t-tests to examine any differences in means between the two groups of children.

Next, to estimate the impact of parental migration on children’s myopia and eyeglasses ownership, we used the following model:

1 $${y}_i={\beta}_0+{\beta}_1{M}_i+{\beta}_2{X}_i+{\varepsilon}_i$$

The analysis is based on a linear probability model (LPM) since the coefficients of this model directly reflect marginal effect and are easy to interpret. Where, *y* is a binary indicator for the myopia status or eyeglasses ownership of student *i* and *M*_*i*_ is a dummy variable that denotes whether there is a migratory parent for student *i* or not. Specifically, if either the father or the mother is a migrant, then *M*_*i*_ equals 1; otherwise, *M*_*i*_ is 0. The coefficient *β*_1_ is the coefficient for parental migration, in which we are interested, and measures the marginal impact of parental migration on children’s myopia or eyeglasses ownership. The vector X is a vector of control variables and *ε*_*i*_ is a random error term. Here, *i* represents each of the observations.

However, if unobserved confounding factors between parental migration and LBC vision health exist (e.g., parent’s vision health), which would lead to a problem of endogeneity. Our DWH (Durbin-Wu-Hausman) test showed that *P* value was less than 0.01 in both the full and myopic samples, indicating that parents’ migrant status was indeed endogenous. To infer causation between parental migration and children’s vision health, we apply the instrumental variable (IV) estimation to address this problem and our main results are based on an IV-LPM model.

It is important to discuss how we choose the IVs. The IVs should be significantly correlated with *M*_*i*_, but do not correlate with *ε*_*i*_. Inspired by Zhao et al. (2014) and Ao et al. (2017), one possible IV is migration probability at the class level [6, 29]. We construct a variable which measures the proportion of parental migrants in the class level as our main IV. The rationale for our choice is as follows. On the one hand, current studies show that social networks are closely related to the migration behavior of household members. Therefore, for households in a class with a richer migration experience, their members will have a higher probability of migration [30, 31]. On the other hand, the migration probability at the class level is presumed to not directly affect myopia or eyeglasses ownership of a child at the individual level. To further verify the validity of the IV, in the latter section, we not only test whether our IV is weak; but also construct a new IV of migration probability at the school level for robustness check.

We adjust standard errors for clustering at the school level in all regression models. All analyses are performed using Stata 15.1 (Stata Corp., Texas, USA).

## Results

### Vision health and background characteristics

Table 1 shows the descriptive statistics of vision health and background characteristics of the LBC and non-LBC. There were 5398 boys (52.1%) and 4963 (47.9%) girls in our sample. The age range was 11 to 18 years, with an average of 14.23 ± 1.32 years. There were 5346 (51.6%) students in 7th grade and 5015 (48.4%) in 9th grade. Overall, the prevalence of self-reported myopia among rural junior students was 54.6% (5660/10,361), and the rate of eyeglasses ownership among myopic students was 40.7% (2304/5660). This means that more than half of myopic students were still not getting a timely correction.

Specifically, the myopia rate (55.8%) and eyeglasses ownership rate (42.0%) for the non-LBC were higher than for the LBC (51.2 and 36.6%). The results from the t-tests indicate statistically significant differences between the two groups for most of the variables (except for age). LBC were mainly from the central regions, with a higher proportion of school boarding, lower academic score, more siblings, less educated parents, and poorer families.

### Effects of the parental migrantion on children’s vision health

Before we use the instrumental variables to assess the effect of parental migration on children’s vision health, it is necessary to test whether our instrumental variable is weak. If the instrument is weak, the normal distribution provides a poor approximation of the sampling distribution of the instrument estimator, even when it contains a large sample size [32]. Table 2 reports the estimation results for the first stage of the instrumental estimation. Overall, our IV had a significant positive effect on the migrant decision in both the full sample (column 1) and myopic sample (column 2). The estimated coefficients were stable and statistically significant at the 1% level. Moreover, the F test values of the first stage regression were 273.17 and 103.19, which were larger than the usual critical value of 10. Thus, weak IV is not a threat to this study.

**Table 2:** First-stage: Effects of migration probability on parental migration status

Variables	Full Sample	Myopic Sample
	(1)	(2)
Migration probability at class level	0.839***	0.838***
	(0.030)	(0.039)
Child Characteristics	Yes	Yes
Family Characteristics	Yes	Yes
Adjusted R-squared	0.147	0.136
F-statistics	273.17	103.19
N	10,361	5660

The dependent variables are the parental migration status for all the columns. Standard errors, in parentheses, clustered at the school level

*** *p* < 0.01

Table 3 presents estimates of the impact of parental migration on vision health from both LPM and IV-LPM models. The dependent variables are myopia in columns (1) and (2) and eyeglasses ownership in columns (3) and (4). The most important parameters are the coefficients for the variable of migration. Several important results emerge from this table. First, we found that parental migration did not increase the likelihood of myopia of LBC. The coefficient (− 0.306) was negative and significantly different from zero in IV-LPM models (columns 2). This means children with any parent who migrated were less likely to be myopic than those with non-migrated parents.

**Table 3:** Second-stage: Effects of parental migration on LBC’s vision health

Variables	Myopia		Owned Eyeglasses	
	(1) LPM	(2) IV-LPM	(3) LPM	(4) IV-LPM
Any parent migrated (1 = yes; 0 = no)	0.000	−0.306***	− 0.042**	− 0.469***
	(0.013)	(0.072)	(0.016)	(0.079)
**Child Characteristics**				
Age (years)	0.013	0.012	−0.004	−0.005
	(0.008)	(0.007)	(0.009)	(0.009)
Boys (1 = yes; 0 = no)	− 0.091***	− 0.085***	0.014	0.019
	(0.010)	(0.011)	(0.015)	(0.016)
9th grade (1 = yes; 0 = no)	0.117***	0.110***	0.095***	0.089***
	(0.021)	(0.022)	(0.027)	(0.026)
Number of siblings	−0.035***	− 0.033***	−0.028***	− 0.028***
	(0.008)	(0.008)	(0.010)	(0.011)
School boarding (1 = yes; 0 = no)	−0.039*	−0.012	0.008	0.047**
	(0.022)	(0.023)	(0.023)	(0.022)
Academic score (standardized)	0.036***	0.033***	0.039***	0.037***
	(0.006)	(0.006)	(0.008)	(0.008)
**Family Characteristics**				
Father educational attainment (years)	0.008***	0.006**	0.008**	0.006*
	(0.003)	(0.003)	(0.003)	(0.003)
Mother’s educational attainment (years)	0.002	0.000	0.015***	0.013***
	(0.002)	(0.002)	(0.003)	(0.003)
Family economic condition:				
Average	0.033**	0.023*	0.062***	0.046***
	(0.013)	(0.013)	(0.017)	(0.017)
Rich	0.037	0.026	0.152***	0.148***
	(0.027)	(0.029)	(0.037)	(0.037)
Livingarea				
Central China	−0.091***	−0.030	0.005	0.088**
	(0.032)	(0.036)	(0.039)	(0.041)
Western China	−0.057**	− 0.004	0.082**	0.146***
	(0.027)	(0.034)	(0.032)	(0.029)
Constant	0.338***	0.426***	0.164	0.281**
	(0.105)	(0.101)	(0.131)	(0.133)
N	10,361	10,361	5660	5660

Standard errors, in parentheses, clustered at the school level

*Abbreviations*: *LPM* linear probability model, *IV* instrumental variable

*** *p* < 0.01, ** *p* < 0.05, * *p* < 0.1

Second, the results show that migration impaired the eyeglasses ownership of LBC (columns 3 and 4). Any parental migration significantly decreased the probability of eyeglasses ownership by 4.2% or 46.9%, depending on the model. This result implies that once myopic, it is more difficult for LBC to get timely correction than non-LBC.

Third, after examining the estimates from both models, we found the LPM estimates in columns 1 and 3 were smaller in magnitude compared to the IV-LPM estimates in columns 2 and 4. This underscores the importance of instrumenting for migration as confounders and reverse causality may have biased the LPM estimates downwards.

Among other important factors influencing myopia, we found that students who were female, in 9th grade, had fewer siblings, had a higher academic score, and had parents with higher levels of education were more likely to be myopic (*P* < 0.05). Many other studies have also found these factors [33, 34].

Turning to eyeglasses ownership, myopic students who were in 9th grade, had fewer siblings and had higher scores were more likely to own eyeglasses (*P* < 0.01). Family characteristics were also highly relevant to eyeglass ownership. The coefficients for the educational attainment of the father and the mother were 0.006 (*P* < 0.1) and 0.013 (*P* < 0.01), respectively, and the former was less statistically significant. This implies that well-educated parents were more likely to care for their children’s vision health. Students from wealthier families were more likely to own eyeglasses than the poor ((*P* < 0.01). Moreover, students who lived in school and dwelled in central or western rural China were also more likely to own eyeglasses than their counterparts.

### Robustness check

So far, the results have shown that parental migration holds a mixed effect on students’ vision health. On the one hand, parental migration reduces the likelihood of myopia in children; on the other hand, it also significantly reduces the likelihood of correction for myopic students. We conduct several robustness checks to check if the results from our basic model are robust. The results of these robustness checks are shown in Table 4.

**Table 4:** Robustness checks the impact of parental migration on LBC’s vision health

	IV-Probit	Migration probability at school level	Add county dummies	Random classes	Two periods data	Both parents migrated
	(1)	(2)	(3)	(4)	(5)	(6)
**Panel A: Myopia**						
Any Parent Migrated (1 = yes; 0 = no)	−0.753***	− 0.286***	−0.593***	− 0.284***	− 0.677**	
	(0.162)	(0.074)	(0.198)	(0.084)	(0.341)	
Both Parents Migrated (1 = yes; 0 = no)						−0.387***
						(0.093)
N	10,361	10,361	10,361	8273	3417	10,361
**Panel B: Owned Eyeglasses**						
Any Parent Migrated (1 = yes; 0 = no)	−1.131***	−0.430***	−0.460*	− 0.427***	− 1.42***	
	(0.159)	(0.075)	(0.256)	(0.091)	(0.513)	
Both Parents Migrated (1 = yes; 0 = no)						−0.586***
						(0.109)
N	5660	5660	5660	4500	1738	5660

Each coefficient represents a separate regression, and all regressions include child and family controls. The independent variables are the indicator of any parent migrated for Column (1)–(5) and the indicator of both parents migrated for Column (6). Standard errors, in parentheses, clustered at the school level

*Abbreviations*: *IV* instrumental variable

*** *p* < 0.01, ** *p* < 0.05, * *p* < 0.1

Considering that our dependent variables are dummy variables, we first estimated the IV-Probit model (Column 1), and the results were similar. Second, we replaced the instrument variable with the peer migration rate at the school level to check the robustness of our IV construction (Column 2). The reason for using this instrumental variable was similar to our previous discussion on the use of peer migration rate at the school level. The results shown that F values in the first stage were larger than 10 in both the full sample and myopic sample, and the estimated coefficients of any parent migrated remains virtually unchanged compared with the base model. Third, in Column 3, we added county dummies to control for the county unobserved shocks. This led to a larger coefficient on myopia and a less significant in the impact on eyeglasses ownership (*P* < 0.1).

Fourth, if students are not randomly assigned to classes, this may lead to bias in our instrumental variables. We, therefore, used the randomly sorted sample in the regressions in column 4. We found that the magnitude and statistical significance of the estimated coefficients changed little. Lastly, we replaced our definition of LBC by replacing our independent variable with both parents migrated (compared to no parents migrated or only one parent migrated). Fifth, the follow-up data, including students in 7th grade at baseline, was also available. Therefore, we used two-period data to examine how parental migration affected the vision health of the LBC in Column (5). We found that LBC with any parent migrated during the two periods were significantly less likely to be myopic and less likely to have their myopia corrected. This result was comparable to the findings based on the baseline samples from the 7th and 9th grades. Column (6) reports the estimates using the new definition. As expected, we found a larger impact of parent migration on myopia and eyeglasses ownership.

### Mechanisms

We analyze possible mechanisms in this subsection to better understand how parental migration affects students’ vision health. The first two columns of Table 5 report mechanisms by which parental migration affects the likelihood of myopia; the second two columns report mechanisms by which parental migration affects the likelihood of eyeglasses ownership.

**Table 5:** Mechanism of the impact of parental migration on LBC’s vision health

	Myopia			Owned Eyeglasses		
	Time on outdoor	Time on internet	After-school classes	Watching TV with parents	Parent-child interaction	Parents are the first kit
	(1)	(2)	(3)	(4)	(6)	(5)
Any Parent Migrated	0.288*	0.166	−0.280***	−0.541***	−0.325***	− 0.182***
	(0.165)	(0.214)	(0.067)	(0.076)	(0.054)	(0.048)
F statistic of the first stage	269.14	273.17	271.75	130.59	273.17	272.83
N	10,288	10,347	10,042	10,172	10,361	10,300

Outdoor time and Internet time measure the time (in hours) students spend on outdoor sports activities and the internet or playing games on weekdays, respectively. The last four mechanism variables are dummy variables. After-school classes was scored 1 if a child attended after-school classes; Watching TV with parents was scored 1 if a child watched TV with parents more than once a week; Parents are the first kit was scored 1 if a child asked help from their parent first when in trouble. Parent-child interaction was scored 1 if parents often actively discussed with children about their worries. These mechanism variables have some missing values. The dependent variables are the myopia indicator in Columns 1–3 and the eyeglasses ownership indicator in Columns 4–5. Standard errors, in parentheses, clustered at the school level

*** *p* < 0.01, * *p* < 0.1

According to the literature, outdoor activities and close work are the main factors affecting myopia. Thus, we first investigated the impact of parental migration on children’s outdoor time, internet time (near work), and attendance at after-school classes (near work). The findings revealed that LBC spent more time outside, the same amount of time on the internet, and were less likely to attend after-school classes, which might explain why LBC were less likely to be myopic (see Column (1)–(3) in Table 5).

As for why the rate of eyeglasses ownership among LBC was lower, it may be because the myopia problem of LBC is neglected due to migration. The first step in correcting myopia with eyeglasses is to discover it . However, because LBC do not live with their parents as much, parents are less likely to detect their children’s myopia problem, let alone provide them with eyeglasses. Thus, we initially tested this mechanism in a life scenario of watching TV with parents because children with uncorrected myopia would sit closer or squint when watching TV so that parents could notice their children’s myopia. Not surprisingly, the results showed that LBC were less likely to watch TV with their parents more than once a week (Column 4 in Table 5). Secondly, we discovered that parents of LBC less frequently actively communicate with their kids (Column 5 in Table 5). These results imply that parents of LBC are less likely to notice their children’s myopia problems.

On the other hand, in addition to parents discovering their children’s myopia themselves, studies have shown that whether or not children inform parents of their myopia problems can also influence the prescription of eyeglasses for correction [35]. Considering that the questionnaire did not directly ask students whether they informed parents about their myopia problems, we made a tentative test on whether parents were the first to ask for help when they needed help. The results showed that compared to non-LBC, the proportion of LBC choosing their parents as the first help provider was lower. In other words, LBC were less likely to inform their parents when they had myopia on time than non-LBC, which led to the failure of LBC to correct their myopia with eyeglasses on time.

### Heterogeneous analysis

We further analyzed the heterogeneous effects of parental migration by adding interaction terms to our instrumental variable model. As shown in Table 6, we found no significant evidence of heterogeneous effects across most student demographic and family characteristics, including gender, grade level, academic achievement, school boarding, and parental education level (Table 6, rows 1–3, rows 5–7). We only found that parental migration made children from families with more siblings less likely to be myopic (Table 6, rows 4, column 1). This may be because LBC with more siblings faces less supervision so that they may spend more time outdoors and less time studying, lowering myopia risk.

**Table 6:** Heterogeneous effect of the impact of parental migration on LBC’s vision health

Variables	(1) Myopia	(2) Owned Eyeglasses
Any parent migrated * Boys	0.045	0.050
	(0.062)	(0.094)
Any parent migrated * 9th grade	0.007	0.072
	(0.094)	(0.129)
Any parent migrated * School boarding	0.133	−0.054
	(0.122)	(0.108)
Any parent migrated * Number of siblings	−0.085**	− 0.079
	(0.043)	(0.071)
Any parent migrated * Academic score	0.035	0.033
	(0.050)	(0.049)
Any parent migrated * Father’s educational attainment	− 0.005	−0.018
	(0.014)	(0.017)
Any parent migrated * Mother’s educational attainment	−0.006	−0.020
	(0.009)	(0.014)
Any parent migrated * Family economic conditions		
Any parent migrated *Average	−0.065	−0.089
	(0.066)	(0.110)
Any parent migrated *Rich	0.357	−0.435
	(0.241)	(0.367)
Control Variables	Yes	Yes
N	10,361	5660

Standard errors, in parentheses, clustered at the school level

** *p* < 0.05

## Discussion

Very few published quantitative studies have measured parental migration’s effects on LBC’s vision health. Based on a nationally representative dataset of China, our empirical analysis not only provides evidence on this topic but also contributes to a more comprehensive understanding of the effects of parental migration on LBC’s health. Taking myopia and eyeglass ownership as indicator variables of vision health, our results show that parental migration has both positive and negative effects on the vision health of LBC.

On the one hand, we found that parental migration reduced the likelihood of myopia in LBC. Some descriptive studies have also found a lower prevalence of myopia among children left behind compared to their counterparts [3, 15]. Moreover, our mechanistic analysis indicates that the low myopia rate among LBC might result from the fact that they spend more time on outdoor activities and less time on near work (e.g., fewer after-school classes). Some other studies have also found that non-LBCs spend more time on homework, after-school reading [16, 36, 37], etc., which may raise their risk of myopia. It should be noted that the prevalence of myopia rate was over 50% for both groups of students, which means that the vision health of all students in rural China needs attention.

On the other hand, parental migration makes it more difficult for LBC with myopia to receive timely correction. Our findings demonstrate that parental migration reduces the likelihood of LBC owning eyeglasses by around 40%, and a set of robustness tests also support this finding. This means that parental migration leads to inequity in correcting myopic left-behind children. Although few studies have examined the influence of parental migration on students’ uptake of eyeglasses, some studies have verified that parental migration lowers left-behind children’s use of health services [4, 38, 39]. A meta-analysis also found that the healthcare needs of children separated from their parents are more likely to be overlooked [28]. According to the findings of our study, the uptake of eyeglasses as a health service is not an exception. Furthermore, we analyzed the possible mechanisms for the low correction rate of myopic LBC. Our empirical findings indicated that because LBC and their parents live apart, myopia in LBC was more difficult to detect by the parents, and LBC were less likely to inform their parents if they were myopic actively.

Considering that parental migration has caused the inequality of myopia correction for LBC, parents of LBC should be more concerned about their children’s visual health and encourage them to share if they cannot see clearly. In addition, a social support network for LBC from other potential providers, including teachers, and other caregivers, is needed to counteract the negative effects of parental migration on children’s myopia correction. Fortunately, research has shown that teachers informing parents about their children’s myopia has a positive effect on myopia correction [35]. It may be helpful to encourage teachers to inform parents when they identify myopia problems among LBC. Moreover, Guan et al. (2018) found that left-behind children who receive an eyeglasses voucher are more likely to redeem it and use it both in the short term and long term [40]. Therefore, policymakers should try increasing the correction rate by providing subsidies to families with LBC.

This study adds some important value to the subject in multiple ways. First, based on representative data, our study enriches the literature on the effects of parental migration, especially in health. In contrast to previous descriptive analysis studies, we assessed causality, which deepens the understanding of the relationship between parental migration and the visual health of LBC. Second, this study also has practical implications for myopia prevention and control in China. In the context of China’s high myopia rate, the findings from this study would be useful for developing myopia control interventions among LBC, especially in terms of timely corrective treatment. Third, our study discusses how parental migration affects LBC’s vision health, which contributes to a better understanding of the effects of parental migration.

The study also has several limitations. First, due to data limitations, we did not investigate the impacts of different durations of parental migration on LBC’s vision health in the short and long run. Future studies should consider this topic carefully. Second, self-report bias may influence our results because myopia and spectacles ownership are self-reported variables. Third, our dataset from CEPS did not collect data on parents’ myopia and eyeglasses wear status, parents’ communication with children about myopia, parents’ attitudes toward wearing eyeglasses, etc. Moreover, the data used in this study was collected in 2013. Future studies using updated datasets with more detailed information may improve the estimations.

## Conclusions

This study was based on a representative dataset from China, which included over 10,000 7th and 9th grades students from junior schools. Using an instrumental variable approach, we found that while parental migration reduced the risk of myopia in LBC, it led to inequity in correction. Given that uncorrected myopia can negatively affect academic performance, mental health, and many other aspects, it is necessary to give more attention to the vision health of LBC. Some public health actions or interventions aimed at eliminating inequalities in the utilization of eye care services for LBC may be worthwhile.

## Data Availability

Database available from the China Education Panel Survey (CEPS) repository, http://ceps.ruc.edu.cn/English/Home.htm.

## Abbreviation

LBC: Left-behind children

## Footnotes

1 Detailed information about the CEPS survey can be retrieved from the CEPS website (Available at http:// ceps.ruc.edu.cn/English/Home.htm).
